# Choroidal imaging features of KIF11-associated retinopathy: expanding the ocular phenotype

**DOI:** 10.1186/s40942-026-00881-8

**Published:** 2026-06-19

**Authors:** Lily C. Farrell, Justin C. Muste, Atharva Hudlikar, Xin Ying Rachel Song, Pearse A. Keane, Rupesh Agrawal, Jose S. Pulido

**Affiliations:** 1 Department of Pathology, Wills Eye Hospital, Sidney Kimmel Medical College Thomas Jefferson University, Philadelphia, PA USA; 2 Sidney Kimmel Medical College, Mid Atlantic Retina, Wills Eye Hospital, Thomas Jefferson University, Philadelphia, PA USA; 3 National Healthcare Group Eye Institute, Tan Tock Seng Hospital, Singapore, Singapore; 4 NIHR Biomedical Research Centre at Moorfields Eye Hospital NHS Foundation Trust, London, UK; 5 Institute of Ophthalmology, University College London, London, UK

**Keywords:** KIF11, Choroid, Pachychoroid, Choroidal vessels, Choroidal vascularity index, Inherited retinal disease

## Abstract

**Background:**

To characterize foveal architecture and choroidal morphology in KIF11-associated retinopathy using multimodal imaging, and to assess for pachychoroid-like features using quantitative choroidal thickness (CT) and choroidal vascularity index (CVI).

**Methods:**

In this retrospective case series, multimodal imaging from two patients (four eyes) with genetically confirmed KIF11-associated retinopathy was analyzed. En-face optical coherence tomography (OCT) images of the macular and inferior peripheral retina were spatially aligned to widefield fundus images to enable fovea-centered spatial mapping. The choroid was segmented and binarized on OCT B-scans to derive quantitative macular CT and CVI measurements, and CT measurements in the mid-peripheral and peripheral retina. Pixel-based measurements were converted to microns using image-scale calibration. Measurements from both eyes were compared with a healthy control.

**Results:**

Both KIF11 patients demonstrated bilateral fovea plana, inferior chorioretinal dysplasia, and inferotemporal vascular dragging. Sub-macular CT showed altered spatial patterning relative to a reference control, with lower overall thickness and increased inter-eye variability. In control eyes, CT demonstrated fovea-centered distribution, whereas patient eyes showed disrupted spatial organization, most pronounced in P1 (OD: R² = 0.41; OS: R² = 0.52). Sub-macular CVI ranged from 58 to 65% and demonstrated increased variability in spatial patterning, with reduced quadratic model fit in KIF11 patient eyes (P1 OS: R² = 0.09). Inferior retinal regions showed diffusely increased CT, with focal maximal thickening immediately temporal to chorioretinal dysplasia, forming a sharp, reproducible transition zone. Beyond this, CT progressively decreased with increasing temporal distance from the atrophic border. The ratio of CT at the transition point relative to distal temporal measurements consistently exceeded 1.0.

**Conclusions:**

KIF11-associated retinopathy may demonstrate regional pachychoroid-like choroidal phenotype with spatially patterned dysplasia, most pronounced adjacent to areas of chorioretinal atrophy. These preliminary findings may implicate choroidal structural and vascular abnormalities as potential contributors to disease pathogenesis and expand the recognized ocular phenotype of KIF11-related retinal disease. Given the exploratory nature and limited sample size of this study, further studies, on a larger sample size, are warranted to further characterize choroidal morphology in KIF11-associated retinopathy.

**Clinical trial number:**

Not applicable.

**Supplementary Information:**

The online version contains supplementary material available at https://doi.org/10.1186/s40942-026-00881-8.

## Introduction

The kinesin-like protein 11 gene **(**KIF11), a member of the kinesin-5 family of motor proteins, is located on chromosome 10q23.33 and encodes Eg5. Eg5 is a microtubule-based, homo-tetrameric motor protein essential for centrosome separation, bipolar spindle formation, and chromosome alignment during mitosis [1]. Beyond its mitotic function, KIF11 also plays an important role in non-dividing cells, including neuronal development, axonal transport, and endothelial cell proliferation. It has been implicated in the organization of tubulin within centrioles of the primary cilium across multiple cell types, highlighting its multifunctional role in both dividing and differentiated cells [2, 3].

Pathogenic variants in KIF11 have been associated with two distinct disease entities: microcephaly with or without chorioretinopathy, lymphedema, or intellectual disability (MCLMR) and familial exudative vitreoretinopathy (FEVR) [4, 5]. Collectively, the retinal manifestations across this spectrum have been termed KIF11-associated retinopathy. The predominant ocular manifestation of KIF11-associated retinopathy is chorioretinal dysplasia, which most commonly manifests bilaterally with a predilection for the inferior mid-periphery of the retina [6, 7]. 

Despite these known manifestations, the underlying pathogenesis of KIF11-related disease remains poorly understood. Given KIF11 expression in vascular endothelial cells, its proposed role in ciliary and vascular organization, and documented retinal vascular phenotype, characterized by a failure of peripheral retinal vascularization, evaluation of choroidal structure may therefore provide important insights into potential vascular contributions to disease pathogenesis [3, 5–7]. However, the role of the choroid in KIF11-associated retinopathy has not been systematically evaluated. Accordingly, this study quantitatively assesses pachychoroid-like features in KIF11-associated retinopathy using choroidal thickness (CT) and choroidal vascularity index (CVI). Through these analyses, we aim to provide preliminary insights into choroidal involvement in KIF11-associated retinopathy, which may help expand the currently recognized ocular phenotype.

## Methods

### Study design and participants

This retrospective observational case series includes two patients (four eyes) with genetically confirmed KIF11-associated disease. Patients were identified through the Wills Eye Hospital ocular genetics database. Inclusion criteria required the availability of high-quality multimodal retinal imaging suitable for quantitative analysis. The study was approved by the institutional review board (IRB) of Wills Eye Hospital with a waiver of informed consent, and all research procedures adhered to the tenets of the Declaration of Helsinki. As no directly comparable normative datasets were identified in the literature, two healthy eyes from an individual with no known ocular pathology were included as internal controls for quantitative comparison. Additionally, to account for the potential confounding effects of chorioretinal scarring on imaging characteristics, two independent eyes with inactive toxoplasmosis chorioretinal lesions were included for exploratory qualitative comparison. These cases were used for illustrative purposes only and were not intended to serve as formal controls. A healthy control (2 eyes) was included for descriptive and contextual comparison only and was not intended to support inferential statistical or biologically definitive conclusions. Choroidal measurements were interpreted cautiously given the known influence of age, axial length, and refractive error on CT and CVI metrics. Axial length and refractive error data were not available and therefore could not be controlled for in the present study.

### Imaging acquisition

All available multimodal retinal imaging was extracted for each eye. Color fundus imaging was acquired using a confocal scanning laser ophthalmoscope (Optos California P200DTx; Optos Inc., Marlborough, MA, USA). Spectral-domain optical coherence tomography (OCT) was performed using the Heidelberg Spectralis system (Heidelberg Engineering, Heidelberg, Germany). Thirty-degree OCT scans were obtained through the foveal region, and fifty-five-degree radial OCT scans were acquired through the mid-peripheral and peripheral retina to capture areas of chorioretinal dysplasia. Similar OCT imaging protocols were applied to a healthy control subject (two eyes). Additionally, OCT B-scans of the right eye of two independent patients with inactive chorioretinal scarring secondary to toxoplasmosis were acquired.

### Imaging analysis

All available multimodal imaging was reviewed to assess for structural abnormalities. Widefield color fundus images (Optos) and corresponding en-face OCT images were spatially aligned using the Fiji (ImageJ) plugin BigWarp [8] (Supplementary Fig. 1) to ensure accurate cross-modal registration. Following alignment, Optos Advance software (Optos Inc., Marlborough, MA, USA) was used to measure the vertical distance from the center of the fovea to the first horizontal OCT B-scan location on the en-face OCT image. Subsequent measurements between OCT B scans were obtained directly from the acquisition parameters of the Heidelberg Spectralis OCT system and used to confirm relative scan positions.

### Choroidal measurements

**Fig. 1:**
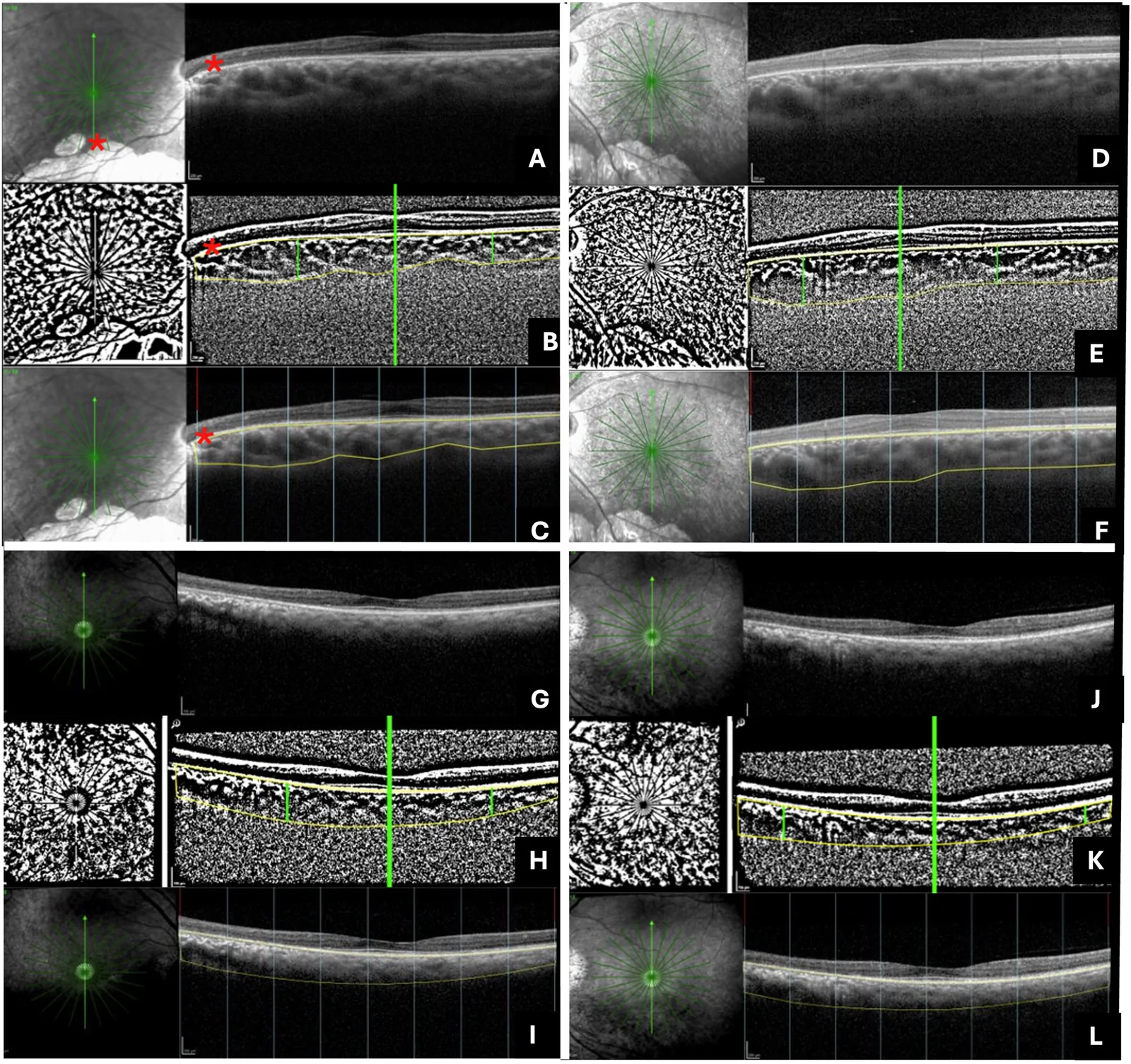
Analysis of sub-macular choroidal morphology. **(A)** Optical coherence tomography (OCT) of the right eye (OD) of patient 1 (P1) demonstrating inferior chorioretinal dysplasia (asterisk). OCT P1 OS (**D**), Patient 2 (P2) OD (**G**), P2 OS (**J**). **(B)** Corresponding binarization and delineation of the region of interest (ROI) is denoted by yellow lines. **(C)** Segmentation of the ROI allowing for choroidal thickness (CT) and choroidal vascularity index (CVI) analysis. Corresponding OCT, ROI delineation, and segmentation are shown for the left eye (OS) of P1 in (**D–F**), patient 2 (P2) OD in (**G–I**), and P2 OS in (**J–L**)

De-identified OCT images were extracted and uploaded to the Comprehensive Ocular Imaging Network (COIN) software to calculate choroidal vascularity index (CVI) [9] and choroidal thickness (CT). Both thirty-degree OCT scans through the foveal region and fifty-five-degree scans through the inferior mid-peripheral to peripheral retina, encompassing regions of chorioretinal dysplasia, were analyzed. The region of interest (ROI), also referred to as the total choroidal area, was defined using the retinal pigment epithelium (RPE)-Bruch’s membrane interface as the superior boundary and the choroidal-scleral junction as the inferior boundary (Fig. 1). These boundaries were identified based on their characteristic hyperreflectivity on OCT. Within regions of chorioretinal dysplasia, ROI was focused on the choroid temporal to this lesion. To minimize inter-observer variability in ROI delineation, images were binarized using the Niblack binarization technique to enhance visualization of these interfaces as continuous pixelated boundaries (Fig. 1). The Niblack binarization technique was selected due to its use of locally adaptive thresholding, whereby image contrast is adjusted according to local pixel intensity rather than a single global threshold [10]. This facilitates improved delineation of choroidal vascular lumina and the choroidal-scleral interface in OCT images with heterogeneous reflectivity. This method has been widely employed in prior choroidal vascularity index (CVI) analyses and was chosen to enhance consistency of ROI segmentation in regions demonstrating abnormal retinal and choroidal architecture. [9] All ROIs and choroidal segmentation were manually delineated by a single reviewer (L.C.F.), with review by a second reviewer (J.S.P.) in cases of ambiguity to improve measurement consistency and minimize observer-dependent variability.

### Quantitative data extraction

Quantitative measurements calculated within the ROI included luminal area (LA), stromal area (SA), total choroidal area (TCA), choroidal thickness (CT), and choroidal vascularity index (CVI). Luminal area represented the total dark pixel area corresponding to choroidal vasculature, while stromal area represented the light pixel area corresponding to interstitial tissue. Total choroidal area was defined as the sum of luminal and stromal areas. CVI was automatically calculated using the built-in COIN software algorithm as the ratio of luminal area to total choroidal area. CT was measured as the vertical distance between the RPE-Bruch’s membrane interface and the choroidal–scleral junction. Identical measurements were performed on OCT images from the healthy control subject for contextual comparison. CT was measured in pixels (px) and subsequently converted to millimeters using image scale calibration (175px/mm), derived from the acquisition software (COIN), and subsequently converted to microns. CT was also assessed using Fiji (ImageJ) to verify measurements.

### Quantitative analysis

Choroidal thickness and CVI measurements were recorded separately for each eye of each patient. Data were organized and analyzed in Microsoft Excel (version 16.106; Microsoft Corp., Redmond, WA). All measurements were referenced to a fovea-centered coordinate system, with the fovea designated as 0 μm, inferior locations assigned negative values, and superior locations assigned positive values. In the sub-macular region, for each eye, second-order polynomial (quadratic) regression models were fitted using Microsoft Excel to characterize the spatial distribution of measurements, with goodness-of-fit assessed using the coefficient of determination (R^2^). The choice of a quadratic fit is based on the natural distribution of choroidal thickness, as in healthy eyes choroidal thickness peaks sub-foveally and tapers in the superior and inferior directions [11, 12]. A quadratic curve naturally captures this progression. In the inferior mid-peripheral and peripheral retina, quantitative measurements from the right and left eyes were combined at the patient level. Of note, no inferential statistical analyses were performed given the exploratory nature and limited sample size of this study, and all modeling should therefore be interpreted as descriptive rather than analytical.

### Transition point ratio analysis

For inferior retinal OCT B-scans, the ROI was set from a pre-defined transition point to the most temporal point of choroid captured on the scan. The transition point was characterized by an abrupt change in choroidal morphology, most commonly from chorioretinal dysplasia to sudden pachychoroid-like appearance. Identification of this transition point was based on investigator assessment of OCT morphology and was therefore inherently subjective. Formal interobserver and intraobserver reproducibility testing was not performed. This ROI was segmented into eight equidistant regions, and choroidal thickness was calculated for each segment. The segmentation into eight equidistant regions was selected based on a pre-configured segmentation function within the COIN software, allowing standardized spatial assessment across the ROI. A ratio was calculated between the segment closest to the transition point and the segment furthest temporal.

Given the rarity of the disease, limited sample size, and exploratory design, analyses were descriptive and hypothesis-generating, with the control used for qualitative and contextual comparison rather than formal statistical inference.

## Results

### Patient Characteristics

**Fig. 2:**
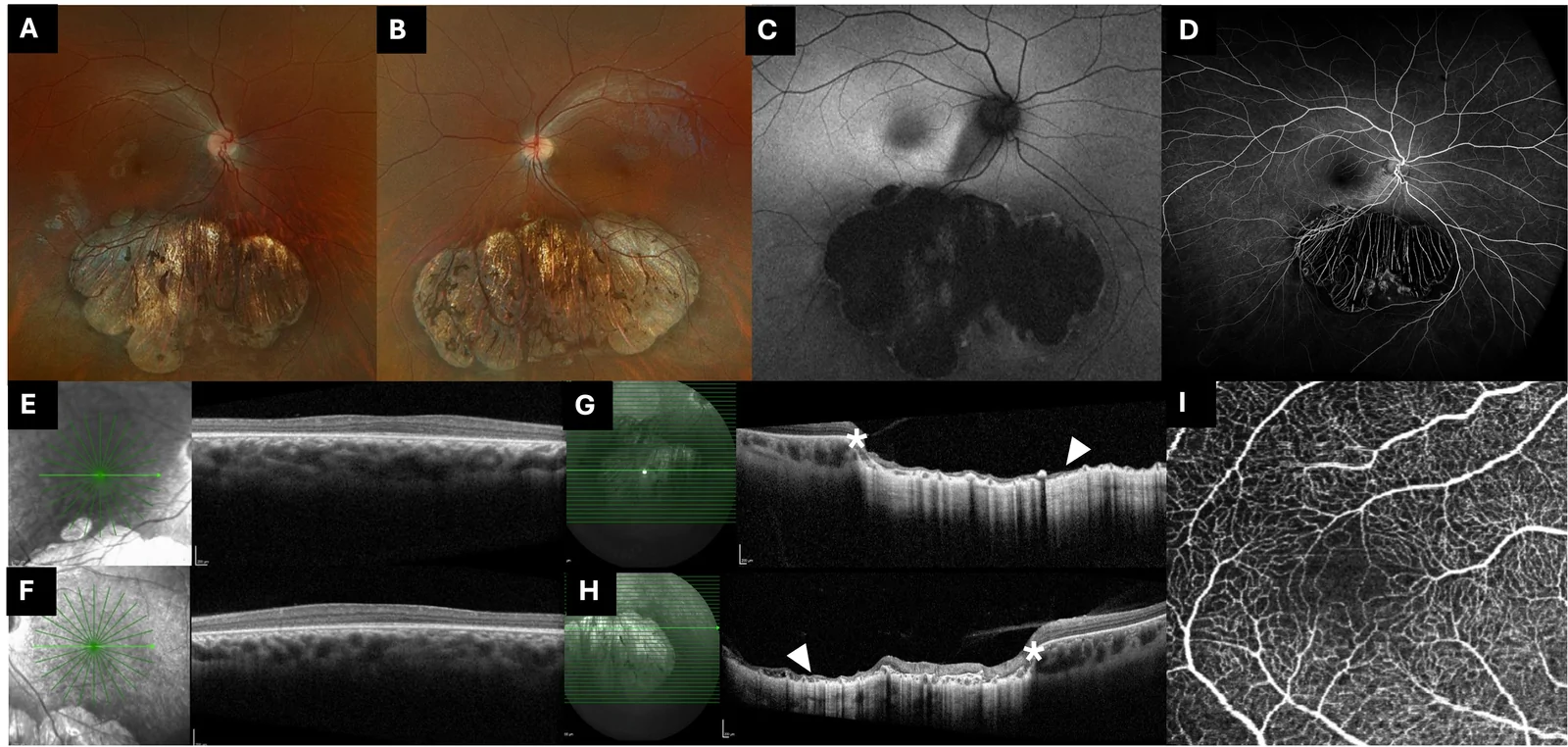
Multi-modal Imaging of Patient 1 with KIF11-associated Retinopathy. Color fundus photograph reveals a large, inferior, scalloped area of chorioretinal dysplasia in both eyes **(A-B)** with corresponding hypo-autofluorescence on fundus autofluorescence (**C**) and window defect on fluorescein angiography (**D**). Optical coherence tomography (OCT) demonstrates fovea plana in both eyes (**E-F**). Peripheral OCT highlights areas of loss of retinal laminations (arrow heads) with adjacent pachychoroid-like changes (asterisk) **(G-H)**. OCT angiography of the right eye highlights an abnormally formed foveal avascular zone **(I)**

**Fig. 3:**
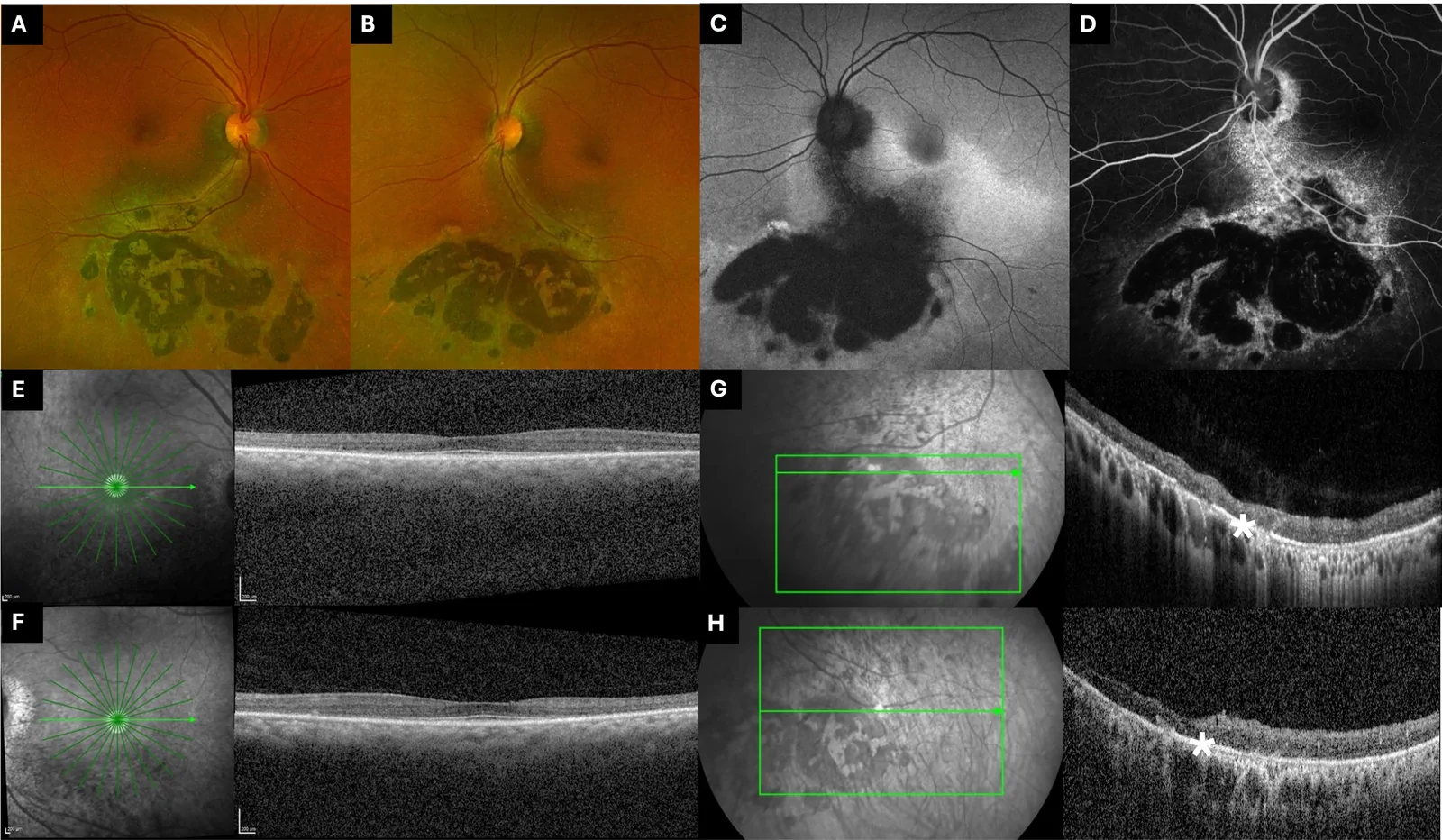
Multi-modal Imaging of Patient 2 with KIF11-associated Retinopathy. Color fundus with inferior chorioretinal dysplasia in both eyes **(A-B)** with corresponding hypo-autofluorescence on fundus autofluorescence **(C)** and window defect on fluorescein angiography (**D**). Optical coherence tomography (OCT) of both eyes **(E-F)** demonstrates elongation of the foveal umbo. Peripheral OCT highlights disruption of normal retinal laminations with associated dysplastic, pachychoroid-like changes (asterisk) in both eyes **(G-H)**

Demographic and clinical features of patient 1 and patient 2 are summarized in Table 1, with multimodal imaging findings presented in Figs. 2 and 3. Both patients had known pathogenic KIF11 variants. The control subject was a healthy 25-year-old female with no known ocular or systemic disease. Both patients exhibited bilateral inferior chorioretinal lesions with inferotemporal vascular dragging on fundus examination, along with fovea plana on OCT B-scans. Systemic features consistent with KIF11-associated disease were observed, including microcephaly in both patients and mild intellectual disability in patient 1.

**Table 1:** Demographics and clinical presentation of KIF11 Patients

	Patient 1 (P1)		Patient 2 (P2)	
Age (y), Sex	14, F		60, F	
Ethnicity	Caucasian		African American	
Medical History	Asthma Psychiatric Disorder Seizures		Arthritis T2DM Thyroid Disease ILD	
Microcephaly	Yes		Borderline	
Head circumference	51 cm*		53 cm*	
Lymphoedema	No		No	
Intellectual disability	Mild		No	
Other family members affected	Yes - Mother and Siblings		No	
BCVA	**OD**	**OS**	**OD**	**OS**
	20/50	20/60	20/30	20/20
IOP	24	23	14	11
Fundus Findings	Inferior scalloped chorioretinal lesion OU Dragged vessels inferotemporally Fovea Plana		Inferior chorioretinal lesions OU Dragged vessels inferotemporally Fovea Plana	
Additional Ocular Findings	Microcornea OU (10 mm)		Gonioscopy: Narrow Angles OU	
Associated Ocular Symptoms	None		Nyctalopia	
Diagnosis	FEVR secondary to KIF11 mutation ^†^		FEVR secondary to KIF11 mutation ^‡^	

y = Years; F = Female; T2DM = Type 2 Diabetes Mellitus; ILD = Interstitial Lung Disease; cm = Centimeters OD = Right Eye; OS = Left Eye; BCVA = Best Corrected Visual Acuity; IOP = Intra-ocular Pressure; OU = Both Eyes; mm = Millimeters; FEVR = Familial Exudative Vitreoretinopathy

*Normal range for age and gender: 53–58.5 cm

† Genetic variant: Deletion of 1.85 Mb within cytogenic band 10q23.33 (chr.10:94211441–96061902) which includes the *KIF11* gene

‡ Genetic variant: heterozygous for *KIF11* c.339del, p.(Phe113Leufs*23)

### Foveal morphology

Both patients demonstrated abnormal foveal architecture on OCT imaging (Figs. 2E–F and 3E–F). In patient 1 (P1), horizontal OCT B-scans revealed an incomplete foveal umbo in the right eye and complete loss of the foveal umbo in the left eye (Figs. 2E–F). Patient 2 (P2) had bilaterally elongated and shallow foveal contour (Figs. 3E–F). OCT angiography of the right eye of P1 demonstrated an abnormally formed foveal avascular zone (FAZ) (Fig. 2I).

### Choroidal morphology: qualitative assessment

Vertical raster OCT through the fovea demonstrated a sub-macular pachychoroid-like phenotype bilaterally in P1, with enlarged vascular lumina inferior to the fovea compared to the superior region (Fig. 1A-F). Although overall choroidal thickness appeared reduced in P2 relative to P1, enlarged vascular lumina were similarly observed inferior to the fovea (Fig. 1G-L). In the inferior retina, temporal to regions of chorioretinal dysplasia, both patients exhibited pachychoroid-like features, including enlarged vascular lumina with abrupt transitions relative to adjacent areas of chorioretinal dysplasia. These dysplastic regions were characterized by loss of normal retinal laminations and increased hypertransmission of light (Figs. 2G-H and 3G-H). Comparable pachychoroid-like choroidal changes were not observed in OCT scans of inactive toxoplasmosis chorioretinal scars in two independent right eyes (Supplementary Fig. 2).

### Quantification of pachychoroid: sub-macular region

**Fig. 4:**
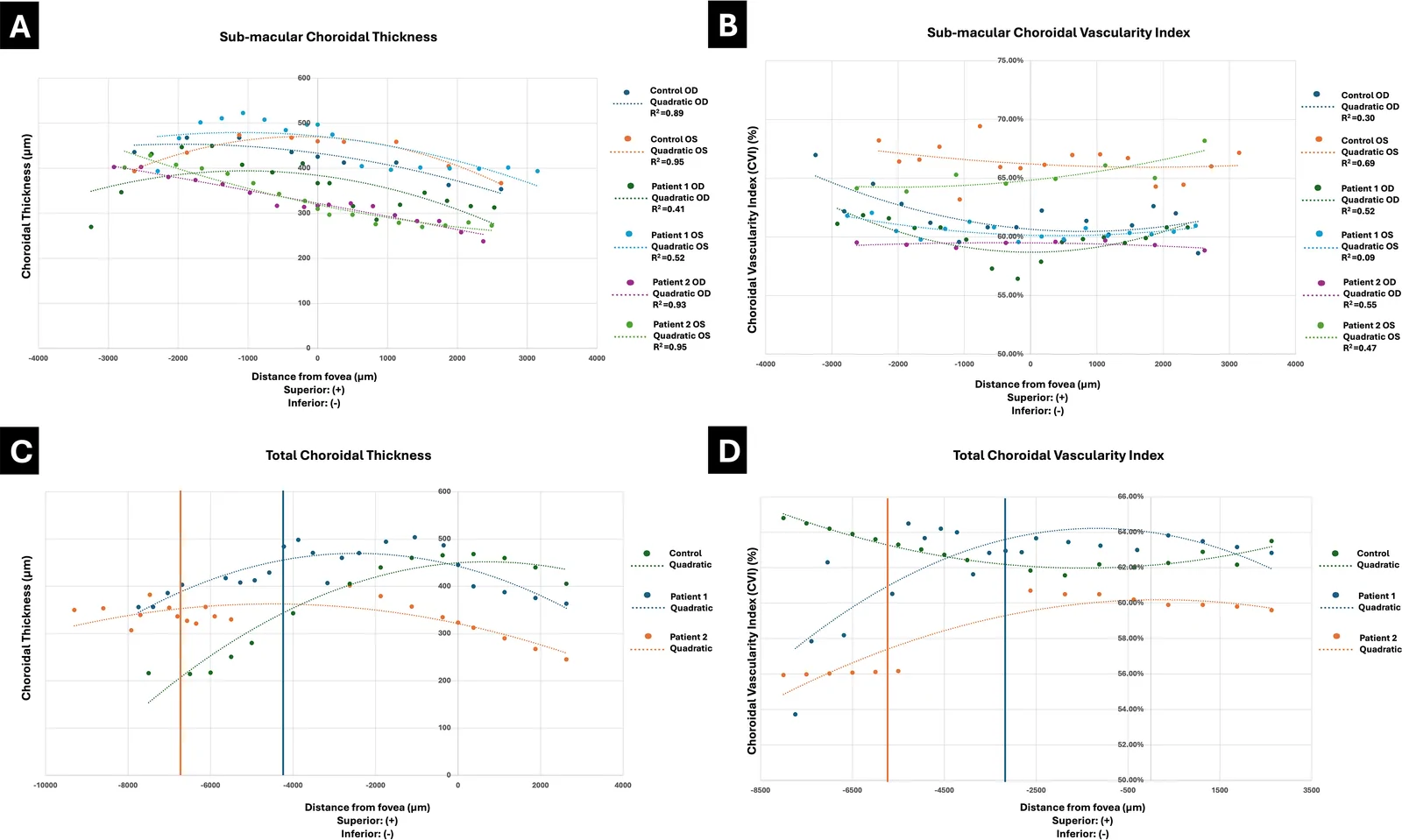
Quantitative Analysis of Sub-macular and Inferior Mid-peripheral and Peripheral Choroid. **(A)** Sub-macular choroidal thickness (CT) at an individual eye level derived from optical coherence tomography (OCT) with corresponding choroidal vascularity index (CVI) measurements **(B)**. **(C)** Peripheral CT with the initiation points of the chorioretinal dysplasia in patient 1 (blue vertical line) and patient 2 (orange vertical line) with corresponding CVI measurements **(D)**

Sub-macular CT measurements are shown at the individual eye level (Fig. 4A; Supplementary Fig. 3). Sub-macular CT was evaluated across the fovea-centered coordinate system, without pre-specification of an expected spatial distribution. Sub-macular CT varied with distance from the fovea in both control and patient eyes. In control eyes, CT demonstrated a consistent spatial distribution centered on the fovea. In contrast, both patients showed overall thinner choroidal measurements, with greater inter-eye differences in spatial pattern and variability. P2 retained a relatively consistent distribution whereas P1 showed greater variability (OD: R^2^ =0.41 and OS: R^2^ = 0.52) (Fig. 4A; Supplementary Fig. 4).

Sub-macular CVI measurements are shown at the individual eye level (Fig. 4B; Supplementary Fig. 3). CVI was similarly evaluated without prespecified assumptions with respect to spatial patterning. CVI varied with distance from the fovea in both control and patient eyes. Patient eyes demonstrated variable spatial patterns with patient 2 demonstrating relatively consistent spatial distribution in both eyes (OD: R^2^ = 0.55; OS: R^2^ = 0.47) while patient 1 exhibited inter-eye asymmetry (OD: R^2^ = 0.52; OS: R^2^ = 0.09).

### Quantification of pachychoroid: inferior mid-peripheral and peripheral retina

Inferior CT measurements showed pronounced spatial variability beyond the initiation point of chorioretinal dysplasia (Fig. 4C). In both patients, CT values within dysplastic regions exceeded those observed in the control at corresponding distances. Maximal CT measurements were recorded within 2000 μm of the lesion initiation point with substantial spatial variation in CT also observed. Despite overall increased CT in the inferior mid-peripheral and peripheral retina, a consistent decline in thickness was observed with increasing inferior distance from the fovea in P1, decreasing from 498 μm at − 3872 μm to 356 μm at − 7744 μm (Fig. 4C). Mid-peripheral CT data was unavailable in P2. CVI increased following lesion initiation in P1 before declining beyond approximately − 5000 μm (Fig. 4D). In P2, CVI values beyond the initiation of the dysplastic region showed minimal variability, with a mean of 56.07% (SD: 0.09) across a 2500 μm span extending inferiorly from the lesion initiation point (Fig. 4D).

### Transition point

**Fig. 5:**
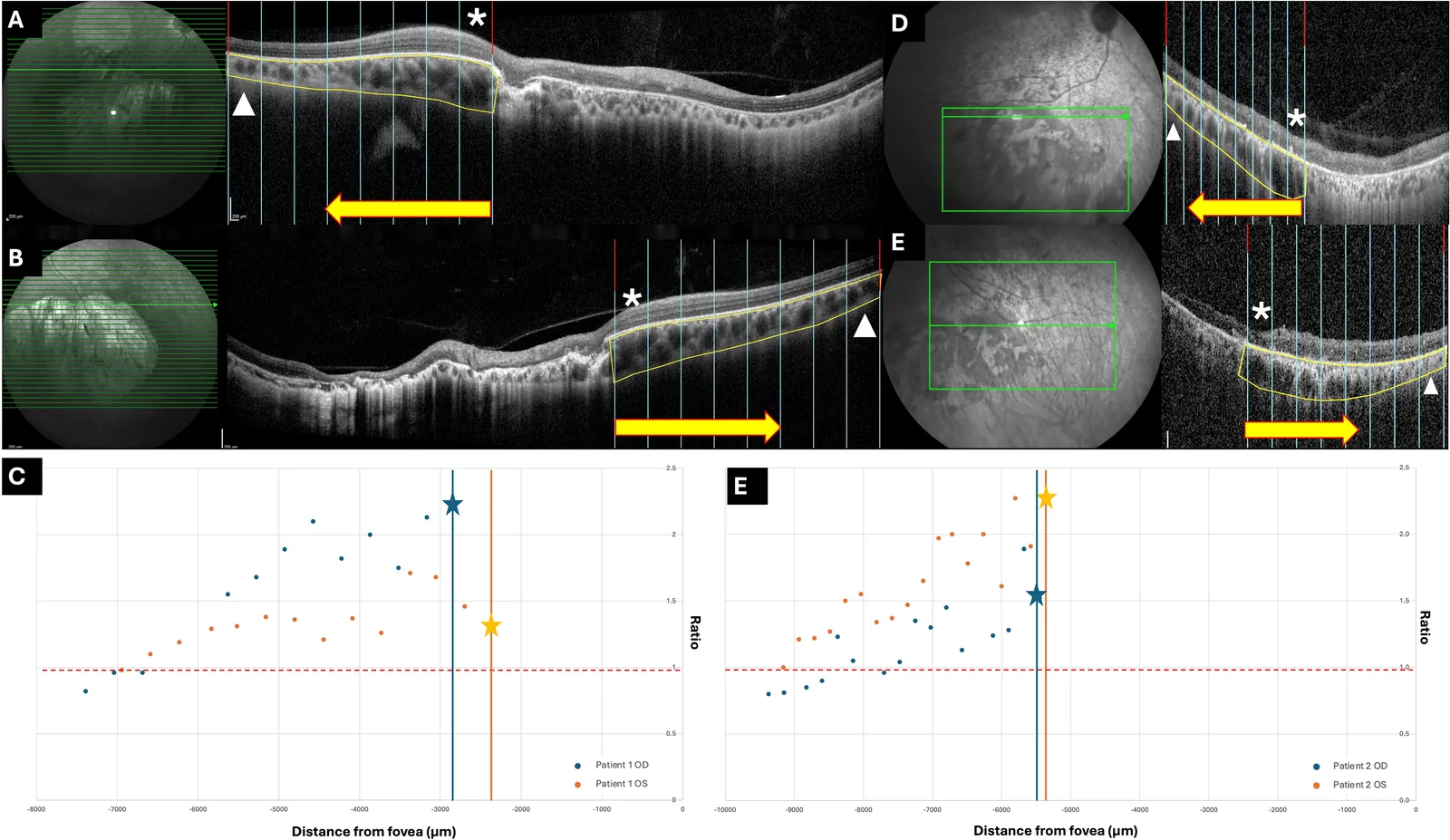
Ratio assessment of choroidal thickness adjacent to chorioretinal dysplasia. In patient 1, the region of interest (ROI) adjacent to chorioretinal dysplasia was segmented into eight equidistant sections in the right (**A**) and left (**B**) eyes. Choroidal thickness (CT) adjacent to the dysplastic border (asterisk) was expressed as a ratio relative to the most temporal measurement (arrowhead) and mapped according to distance from the fovea (**C**). Corresponding analysis was performed in patient 2 in the right (**D**) and left (**E**) eyes, with ratios mapped in (**F**). Blue and orange vertical lines denote the initiation point of chorioretinal dysplasia in the right and left eyes, respectively

For the purposes of this paper, a transition point was taken to be the observable change between normal and pathologic retina. P1 demonstrated a transition point in choroidal morphology immediately temporal to the region of chorioretinal dysplasia that was consistently observed across sequential B-scans extending inferiorly (Fig. 5A-B). CT was maximal at the transition point and progressively declined with increasing temporal distance from the lesion. A similar morphological transition was observed in P2; however, in this case, the transition occurred within the dysplastic region itself (Fig. 5D-E). Across both patients, the ratio of CT at the transition points relative to the most temporally distant measurement consistently exceeded 1.0, with higher ratios proximally with progressive reduction with increasing inferior distance (Fig. 5C-F).

## Discussion

Pathogenic KIF11 variants are classically associated with microcephaly with or without chorioretinopathy, lymphedema, or intellectual disability (MCLMR), a syndrome characterized by defective lymphatic and vascular development. They also account for approximately 5–9% of familial exudative vitreoretinopathy (FEVR) cases characterized by abnormal retinal vasculature. Unlike the peripheral vascular abnormalities and increased straightening of peripheral vessels (ISPV) typical of other FEVR-causing genes, the dominant ocular phenotype associated with KIF11 mutations is bilateral chorioretinal dysplasia, with a predilection for the inferior retina [7]. KIF11-associated disease demonstrates substantial genotypic and phenotypic heterogeneity. In this retrospective observational case series, we expand the recognized phenotype by identifying novel abnormalities in both foveal architecture and choroidal morphology which may be suggestive of a pachychoroid-like phenotype.

In our cohort, we observed abnormal foveal architecture including fovea plana and, in one case, an abnormally formed foveal avascular zone (FAZ). Fovea plana is characterized by persistence of inner retinal layers at the foveal center, a shallow or absent foveal pit, and increased superficial capillary plexus vascular density with a reduced or absent FAZ [13–15]. Normal foveal development involves centrifugal displacement of the inner retinal layers and is closely linked to retinal vascular development [14, 15]. The FAZ forms prior to foveal umbo formation and is a developmental prerequisite for foveal umbo development through both molecular signaling and mechanical mechanisms [16]. Given the developmental interdependence between retinal vasculature and foveal specialization, vascular dysregulation associated with KIF11 mutations could plausibly contribute to abnormal foveal maturation [13–17]. However, this mechanistic relationship cannot be confirmed in the present study and remains speculative. Nevertheless, the presence of fovea plana and abnormal FAZ morphology suggests that careful evaluation of foveal architecture should form part of the clinical assessment of patients with suspected KIF11-associated retinopathy.

In addition to foveal abnormalities, we identified distinct choroidal structural changes that may be suggestive of a pachychoroid-like appearance. Although a universally accepted definition is lacking, pachychoroid is typically characterized by diffuse or focal choroidal thickening with relative attenuation of the inner choroid (choriocapillaris and Sattler’s layer) and dilatation of Haller’s layer (pachyvessels). Quantitative assessment typically relies on choroidal thickness (CT) and, more recently choroidal vascularity index (CVI), with CVI considered less susceptible to physiological variation related to age, sex, and axial length. ^18,19^

Quantitative choroidal analysis in our cohort demonstrated increased CT and CVI abutting areas of chorioretinal dysplasia. Notably, choroidal morphology differed on either side of the dysplastic lesion, with absence of pachychoroid-like morphology nasal to the dysplastic region, suggesting regional specificity rather than generalized thickening. The concurrent elevation in CT and CVI may suggest vascular engorgement rather than stromal expansion as a contributor to choroidal thickening [18–20]. Comparable pachychoroid-like changes were not observed adjacent to inactive chorioretinal toxoplasmosis scars, which may support an argument against a nonspecific reactive response to chorioretinal injury. Collectively, these findings may suggest the presence of pachychoroid-like choroidal alterations associated with KIF11-related dysplastic retinal changes.

Sub-foveal CT in our cohort exceeded published normative ranges, and extra-foveal CT was > 50 μm greater than sub-foveal CT in areas adjacent to dysplasia, a pattern that may be consistent with extra-foveal pachychoroid [20]. While these findings may suggest localized pachychoroid-like choroidal alterations associated with KIF11-related dysplastic retinal changes, comparisons with published normative values should be interpreted cautiously given the absence of age, refractive error, and axial length-matched controls. Additionally, some component of this change may reflect normal variation in CT from the macula to the peripheral retina. Data regarding normal inferior CT distribution are variable, with one study in a healthy Iranian cohort reporting a CT range of 85–481 μm at 3000 μm inferior to the fovea [11]. Other authors reported that inferior peripheral CT decreases by up to 64.5% relative to the macular CT in healthy eyes [21]. CT values of approximately 264 μm at 6000 μm inferior to the fovea have also been reported in healthy eyes [22]. In the absence of standardized peripheral CT metrics, contextualizing our CT findings remains challenging.

Spatial analyses of CVI have similarly demonstrated regional variation. Sub-foveal CVI (SF-CVI) in our KIF11 cohort fell within the accepted physiologic range (65.61 ± 2.33%).^18^ Prior ETDRS-based mapping studies have shown higher CVI centrally compared to the peripheral macula (67.08% vs. 65.68%, *p* = 0.01).^19^ Alanazi et al. reported decreasing CT with a relative increase in CVI as distance from the fovea increased, a pattern also observed in our control (Fig. 4) [23]. Thus, the disproportionate CT and CVI elevation adjacent to dysplasia in our KIF11 patients may represent localized choroidal alterations, although differentiation from physiologic variation is limited by the absence of matched controls. Together, these structural findings raise the possibility that KIF11 may influence choroidal vascular organization or regulation. However, in the absence of age, refractive error, and axial length-matched controls, interpretation remains exploratory.

The exact mechanism of KIF11-associated retinopathy remains poorly understood and KIF11 is not expressed in photoreceptor or retinal pigment epithelial (RPE) cells (Supplementary Fig. 4) [24]. However, the biological relevance of KIF11 in vascular development is supported by its co-expression with FLT4 (VEGFR-3), a key regulator of endothelial proliferation and lymphangiogenesis. FLT4 is expressed throughout the choriocapillaris and choroidal vasculature (Supplementary Fig. 4) [25]. Experimental work by Ogmen et al. demonstrated that KIF11 haploinsufficiency downregulates VEGFR3 expression in lymphatic endothelial cells, establishing a potential relationship between KIF11 dysfunction and impaired lymphatic and vascular channel formation [26]. Despite the absence of conventional lymphatic drainage of the retina, the choroid has been shown to have structural and phenotypic lymphatic-like structures [27]. Given this emerging evidence, KIF11 dysfunction could plausibly impair vascular channel formation and fluid homeostasis within the choroid. Such dysregulation may explain the regional vascular congestion (pachychoroid-like changes) adjacent to zones of vascular insufficiency (leptochoroid), generating the abrupt morphologic transitions observed in our patients [4, 26]. However, these proposed mechanisms remain speculative and cannot be confirmed by the present imaging-based study.

These findings may suggest that choroidal imaging biomarkers, particularly regional CVI and CT gradients, may serve as adjunctive tools in the phenotypic characterization and early detection of KIF11-associated disease.

### Strengths and limitations

To our knowledge, this represents the first quantitative evaluation of choroidal architecture in KIF11-associated retinopathy. Several limitations should be acknowledged. First, the small sample size, reflecting the rarity of KIF11-associated disease, limits generalizability; therefore, these findings should be considered hypothesis-generating and require validation in larger cohorts. While limited in size, this study represents a high-resolution phenotypic characterization using spatially registered multimodal imaging. Second, the retrospective design limited uniform acquisition of peripheral imaging, and quantitative interpretation was constrained by the absence of standardized normative data for peripheral choroidal thickness. Third, CT and CVI are structural surrogate metrics and do not directly assess choroidal perfusion or vascular flow, limiting mechanistic inference. Given the known influence of age, axial length, and refractive status on choroidal thickness measurements, the absence of matched controls and biometric adjustment substantially limits interpretation of absolute CT differences. Accordingly, comparisons with the control subject should be interpreted as contextual and descriptive rather than inferential or biologically definitive. In addition, manual ROI delineation, despite systematic application, may introduce observer-dependent variability. Identification of the transition point was based on qualitative OCT assessment and therefore subject to observer interpretation. Formal interobserver and intraobserver reproducibility testing was not performed. Finally, the proposed mechanistic link between KIF11-mediated endothelial dysfunction and regional pachychoroid-like morphology remains inferential, as direct functional and longitudinal data were not available.

Future studies should aim to build upon these findings through multimodal and longitudinal approaches. In particular, incorporation of optical coherence tomography angiography (OCTA) would enable direct assessment of choroidal and retinal perfusion, helping to distinguish structural from functional vascular alterations in KIF11-associated disease. Longitudinal imaging studies are also warranted to evaluate the temporal evolution of choroidal changes and their relationship to disease progression and visual outcomes. In addition, the application of artificial intelligence–based spatial mapping techniques may facilitate automated, high-resolution quantification of regional choroidal variation, allowing for more robust phenotypic classification and potential biomarker development. Finally, larger multicenter studies integrating detailed genotype–phenotype correlations will be essential to determine whether specific KIF11 variants are associated with distinct choroidal or retinal imaging signatures, thereby advancing precision diagnostics in this rare inherited retinopathy.

## Conclusions

Taken together, these observations may suggest that fovea plana and pachychoroid-like choroidal alterations in KIF11-associated retinopathy may reflect shared vascular developmental or endothelial abnormalities involving both the retinal and choroidal vasculature [26, 28]. These preliminary findings raise the possibility that choroidal structural and vascular changes could potentially contribute to disease pathogenesis and expand the recognized ocular phenotype of KIF11-associated retinopathy. However, given the exploratory nature and limited sample size of this study, further investigation is required to validate these observations and better characterize choroidal morphology in this condition. These findings additionally highlight the need for standardized and reproducible choroidal structural analysis in future studies.

## Supplementary Information

Below is the link to the electronic supplementary material.


Supplementary Material 1: Supplementary Fig. 1: Utilization of the bigwarp function on ImageJ. Color fundus photo of the right eye of patient 1(A) and en-face OCT image (B) superimposed using bigwarp function on ImageJ (C). Color fundus photo of the right eye of patient 2 (D) and en-face OCT image (E) superimposed using bigwarp function on ImageJ (F).



Supplementary Material 2: Supplementary Fig. 2: Optical coherence tomography (OCT) of two patients with inactive toxoplasmosis chorioretinal scars. OCT scans with an oblique cut through the inactive chorioretinal scar highlighting chorioretinal atrophy (asterisk) and corresponding adjacent choroid (arrowhead) in the right eye of 2 independent patients (A & B).



Supplementary Material 3: Supplementary Fig. 3: Quantitative Analysis of sub-macular choroidal thickness and choroidal vascularity index at an individual eye level. Sub-macular choroidal thickness of the right and left control eye (A), patient 1 (B) and patient 2 (C). Sub-macular CVI of the right and left control eye (D), patient 1 (E) and patient 2 (F).



Supplementary Material 4: Supplementary Fig. 4: Color heatmaps demonstrating gene expression within the retina and choroid. (A) Lack of KIF11 gene expression in the RPE or photoreceptor cells. (B) Expression of FLT4 (VEGFR3) in the choriocapillaris and larger choroidal arteries and veins.


## Data Availability

The datasets used and/or analyzed during the current study are available from the corresponding author on reasonable request.

## Abbreviations

CT: Choroidal thickness
CVI: Choroidal vascularity index
FAZ: Foveal avascular zone
FEVR: Familial exudative vitreoretinopathy
LA: Luminal area
MCLMR: Microcephaly with or without chorioretinopathy, lymphedema, or intellectual disability
OCT: Optical coherence tomography
OCTA: Optical coherence tomography angiography
ROI: Region of interest
RPE: Retinal pigment epithelium
SA: Stromal area
TCA: Total choroidal area
